# Pattern of radiation-induced thyroid gland changes in nasopharyngeal carcinoma patients in 48 months after radiotherapy

**DOI:** 10.1371/journal.pone.0200310

**Published:** 2018-07-09

**Authors:** Zhixiong Lin, Zhining Yang, Binghui He, Dangdang Wang, Xiaoyin Gao, Shing-yau Tam, Vincent Wing Cheung Wu

**Affiliations:** 1 Cancer Hospital, Shantou University Medical College, Shantou, China; 2 Department of Health Technology and Informatics, Hong Kong Polytechnic University, Hung Hom, Kowloon, Hong Kong SAR; North Shore Long Island Jewish Health System, UNITED STATES

## Abstract

**Objectives:**

Radiation-induced hypothyroidism is the most common thyroid disorder after radiotherapy in nasopharyngeal carcinoma (NPC) patients. This study evaluated the pattern of radiation-induced thyroid gland changes in 48 months after radiotherapy in NPC patients and the association of hypothyroidism incidence with thyroid dose.

**Methods:**

Fifty-six NPC patients treated by intensity modulated radiotherapy in 2013 were recruited. All patients received baseline thyroid hormones (fT3, fT4 and TSH) tests and CT scan before radiotherapy. Repeated measures of the thyroid hormones and gland volume were performed at 3, 6, 12, 18, 24, 30, 36 and 48 months after treatment. Trend lines of the thyroid volume and hormone level changes against time were plotted. The incidence of hypothyroidism patients and its relationship with the dose were also evaluated.

**Results:**

The mean thyroid volume followed a decreasing trend after radiotherapy, reaching a minimum (-39.8%) at 30 months and slightly increased afterward. The fT4 level followed a similar pattern with its mean value dropped by 21.5% at 30 months and became steady after 36 months. TSH level showed gradual rise from just after radiotherapy, reaching a peak at 24 months and became relatively steady after 36 months. The incidence of hypothyroidism increased to a maximum at 24 months (28.6%) and dropped afterwards. Thyroid D_mean_ and D_50_ were significantly correlated with hypothyroidism incidence in 12 to 30 months (ρ > 0.40, p < 0.05).

**Conclusion:**

The patterns of radiation induced thyroid volume shrinkage and fT4 level reduction were similar, with both of them showed decreasing trend from 0 to 30 months. The thyroid volume and function reached a relatively steady state after 36 months. The incidence of hypothyroidism increased up to 24 months and its frequency was associated with the thyroid dose.

## Introduction

In radiotherapy of nasopharyngeal carcinoma (NPC), bilateral neck irradiation is necessary since neck lymphadenopathy has a high incidence (75–80%)[1]. For conventional radiotherapy treatment, an anterior cervical field is used to treat the whole neck down to the supra-sternal notch, while for intensity modulated radiotherapy (IMRT), the planning target volume of the neck lymph nodes usually includes the nodal level IV, which is adjacent to the thyroid gland. Since currently no dose constraint is applied to the thyroid gland during planning, both techniques may deliver substantial dose to the thyroid gland ranging from 54 to 66 Gy.

Thyroid gland is a major endocrine organ which produces thyroid hormones for maintaining normal body metabolism. Radiation induced thyroid disorders have been reported in post-radiotherapy head and neck cancer patients, such as short-term thyroiditis and long-term hypothyroidism [2, 3]. Radiation-induced thyroiditis is considered as the primary damage to the thyroid gland when the follicular epithelial cells and blood vessels are damaged leading to hypothyroidism as late effect [3]. However, the actual mechanism of radiation-induced thyroid damage is still uncertain and other causes including autoimmune reactions and parenchymal cell damages have also been reported [4, 5]. Common symptoms of hypothyroidism include fatigue and weakness, cold intolerance, cognitive dysfunction, dry skin, constipation and depression, which can affect the quality of life of the post-radiotherapy patients [6].

Radiation induced hypothyroidism is the most common post-radiotherapy thyroid complications with an average incidence of 20–45% and 19–26% in head and neck cancers [5,7–8] and NPC [9–11] respectively. Due to the relatively higher dose to the cervical region in radiotherapy of NPC than those of the head and neck cancers, the risk of hypothyroidism in post-radiotherapy NPC patients was higher [12]. Majority of radiation-induced hypothyroidism are sub-clinical hypothyroidisms which are asymptomatic and can only be detected by thyroid hormone study showing elevated thyroid stimulating hormone (TSH) level. The peak occurrence is 1–3 years after NPC radiotherapy although the onset of radiation induced hypothyroidism can be as long as 10 years after completion of treatment [10,13,14]. Up to now, little is known about the recovery of the thyroid gland after radiotherapy and how this is affected by the radiation dose. This study aimed to study the patterns of radiation-induced thyroid gland changes in NPC patients in first four years after radiotherapy by tracking the thyroid hormones and thyroid gland volume, and evaluate the association of incidence of hypothyroidism with the thyroid gland dose.

## Materials and methods

Fifty-six adult NPC patients (37 male, 19 female, mean age: 48.5 years) treated by IMRT in 2013 were recruited from the Cancer Hospital, Shantou University Medical College. Written informed consent was obtained from the patients before the start of the treatment and ethics approval was obtained from the Research Ethics Committee of Department of Health Technology and Informatics, Hong Kong Polytechnic University. Patients’ identities were masked and replaced by study numbers, and investigators were not able to identify individual subject information after data collection. Each patient underwent computed tomography (CT) of the head and neck region, which were used to compute treatment plan in the radiotherapy treatment planning system (Pinnacle^TM^, Philips, Eindhoven, Netherlands). A seven-beam IMRT plan was delivered using 6 MV photon. Seventy and 66 Gy were prescribed to the planning target volumes (PTVs) of the nasopharynx and neck lymphatics respectively.

The clinical target of the nasopharynx included the nasopharyngeal lesion with margins added to include microscopic involvements, while the clinical target of neck lymphatics included the whole neck covering the lymph nodes at surgical levels II-V. The PTV was created by adding 3 mm to the clinical target. No dose constraints were set for thyroid in routine NPC planning. Dose parameters of thyroid, including maximum dose (D_max_), minimum dose (D_min_), mean dose (D_mean_) and dose of 50% volume (D_50%_) were obtained from dose volume histogram (DVH) generated from the treatment planning system.

For thyroid hormones monitoring, 6 ml of blood was extracted from subjects before radiotherapy with prior consent. Free triiodothyronine (fT3), free thyroxine (fT4) and TSH levels were measured by the electrochemiluminescence method using the Elecsys 2010 analyzer (Hitachi High Technology Corporation, Tokyo, Japan).

All patients were followed-up at regular intervals. Repeated CT scans of the head and neck region and thyroid hormone measurements were performed at 6, 12, 18, 24, 30, 36 and 48 months post-radiotherapy. The thyroid volume obtained from CT and readings of the three thyroid hormones were recorded and the trend lines of each parameter were plotted. The incidences of hypothyroidism including subclinical and overt were recorded based on the thyroid hormone study. Subclinical hypothyroidism was defined as having an elevated TSH level (> 5.6 mIU/L) and normal fT4 level (7.9–14.4 pmol/L), while overt hypothyroidism was defined as having an elevated TSH level (> 5.6 mIU/L) and a lowered fT4 level (< 7.9 pmol/L). The evaluation of the association between dose parameters and hypothyroidism at each time interval was performed using Spearman’s rank correlation test by Statistical Package for the Social Sciences (SPSS) version 22 (IBM Corporation, New York, USA).

## Results

**Fig 1:**
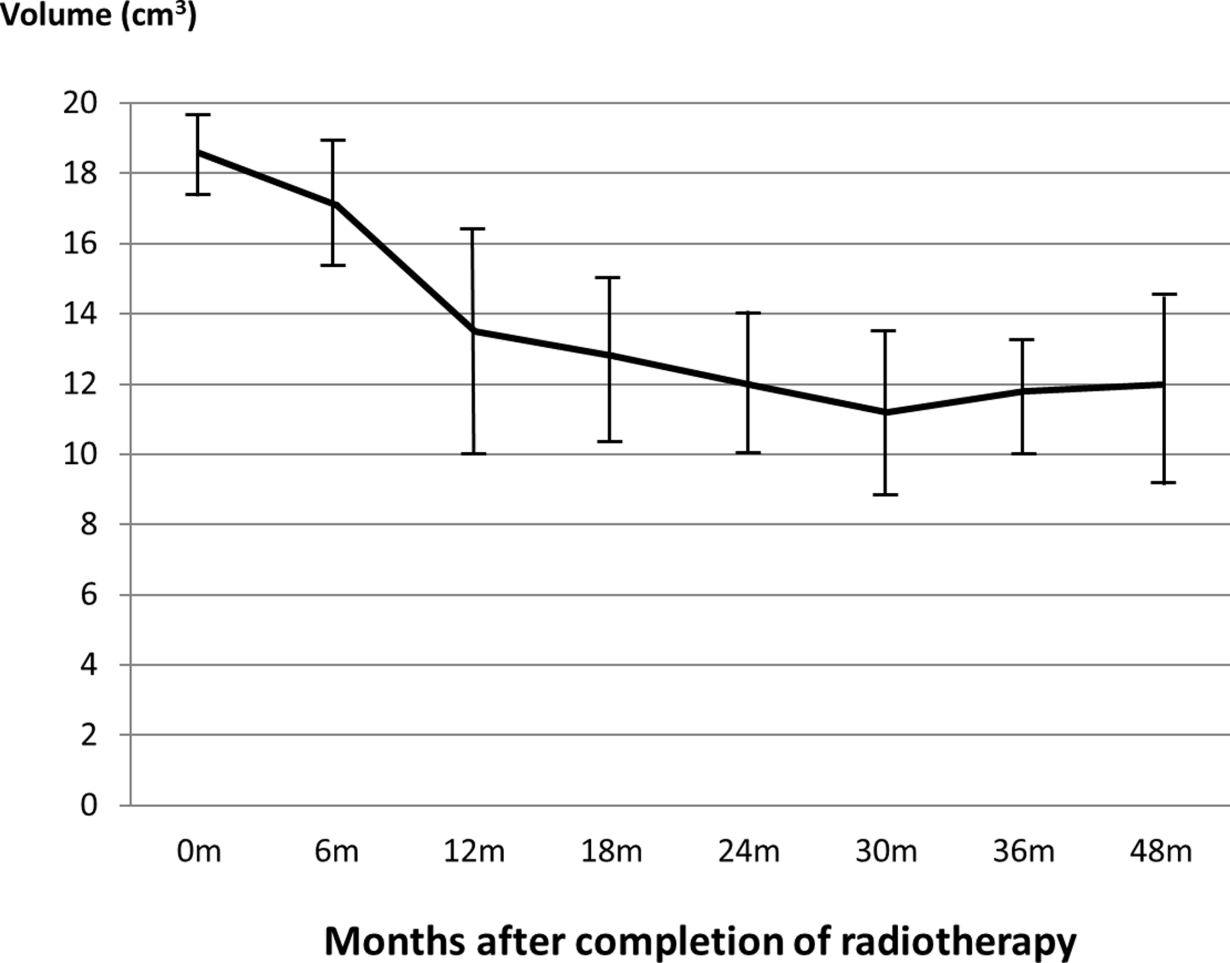
Mean thyroid gland volume from pre-radiotherapy to 48 months after radiotherapy. The error bars represent the standard deviation (SD) of the thyroid gland volume at the specific time interval. 0m = pre-radiotherapy, m = month.

All patients completed the radiotherapy course uneventfully and the patient demographics are displayed in Table 1. The age of the patients ranged from 21 to 71 years with a mean of 48.5 years (median = 51). The thyroid D_mean_ ranged from 23.3 Gy to 58.6 Gy (mean 43.9 Gy) (Table 2). The mean thyroid volume decreased rapidly from 18.6 cm^3^ at pre-radiotherapy by 27.4% at 12 month interval and continued to drop mildly to a minimum of 11.2 cm^3^ at 30 months (-39.8%). After 24 months, the volume of the gland remained relatively steady with variations of less than 5% (Fig 1).

**Table 1:** Patient characteristics (n = 56).

	Number of Patients (Percentage)
Age (year)	
< 30	4 (7.1%)
30–39	6 (10.7%)
40–49	18 (32.1%)
50–59	18 (32.1%)
60–69	9 (16.1%)
>69	1 (1.8%)
Gender	
Male	37 (66.1%)
Female	19 (33.9%)
Stage (AJCC 2002)	
I	0 (0%)
II	4 (7.1%)
III	38 (67.9%)
IVa	13 (23.2%)
Unknown	1 (1.8%)

**Table 2:** Thyroid gland doses received by the NPC patients (n = 56).

Thyroid doses (Gy)	Mean ± SD
D_max_	65.1 ± 2.2
D_min_	23.2 ± 11.7
D_mean_	43.9 ± 9.9
D_50%_	42.5 ± 12.9

**Fig 2:**
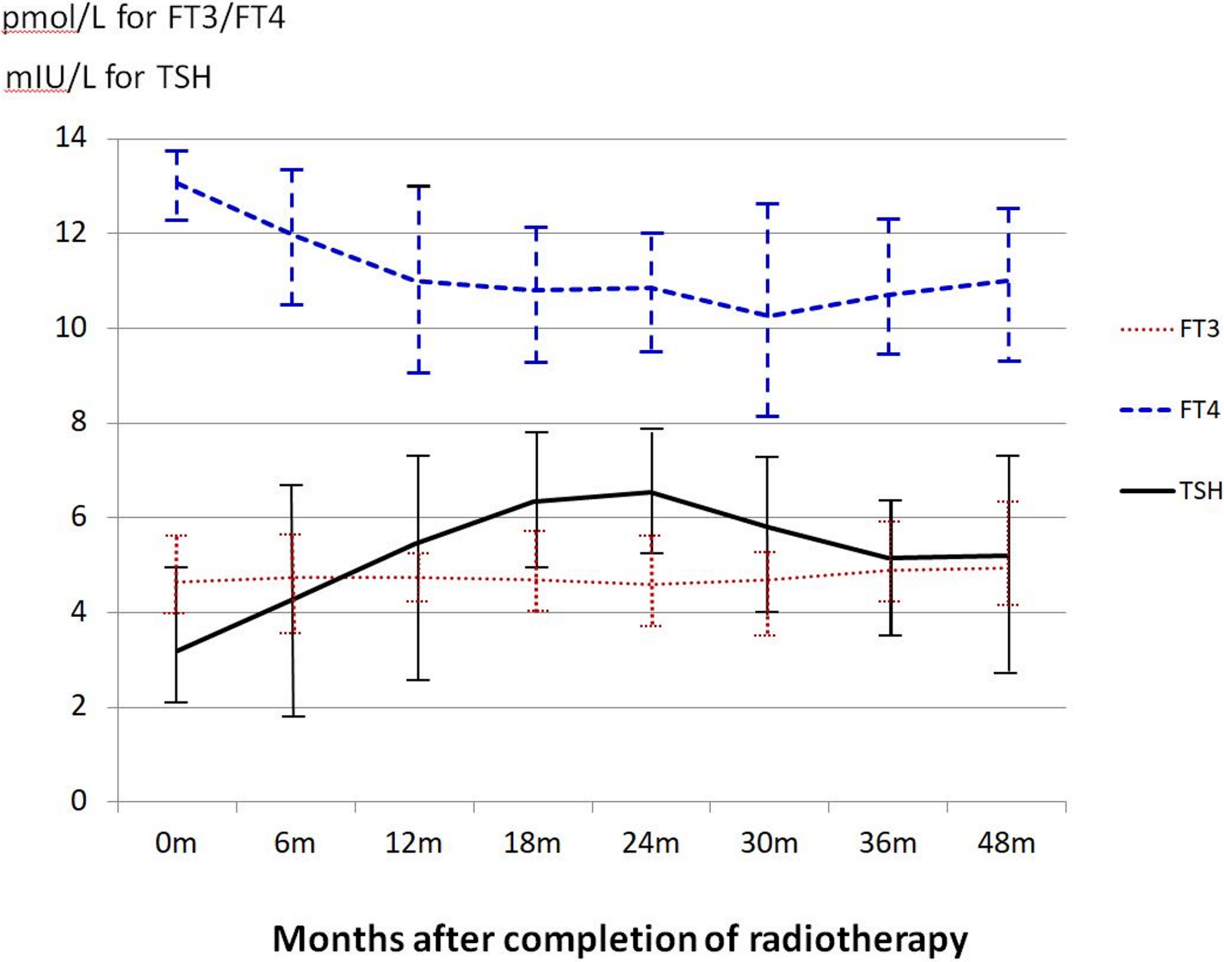
Mean thyroid hormone levels from pre-radiotherapy to 48 months after radiotherapy. The error bars represent the standard deviation (SD) of the hormone levels at the specific time interval. fT3: Free triiodothyronine, fT4: Free thyroxine, TSH: Thyroid-stimulating hormone, 0m = pre-radiotherapy, m = month.

For the thyroid hormones, mean fT3 level remained steady throughout the study period and mean fT4 level decreased from 13.1 pmol/L at pre-radiotherapy to 10.3 pmol/L at 30 months (-23.5%) and became relatively steady after 36 months (10.7 pmol/L). For TSH, the average level showed gradual rise after radiotherapy (0 month), reaching a plateau around 24 months and returned to a steady state 36 months (Fig 2).

**Fig 3:**
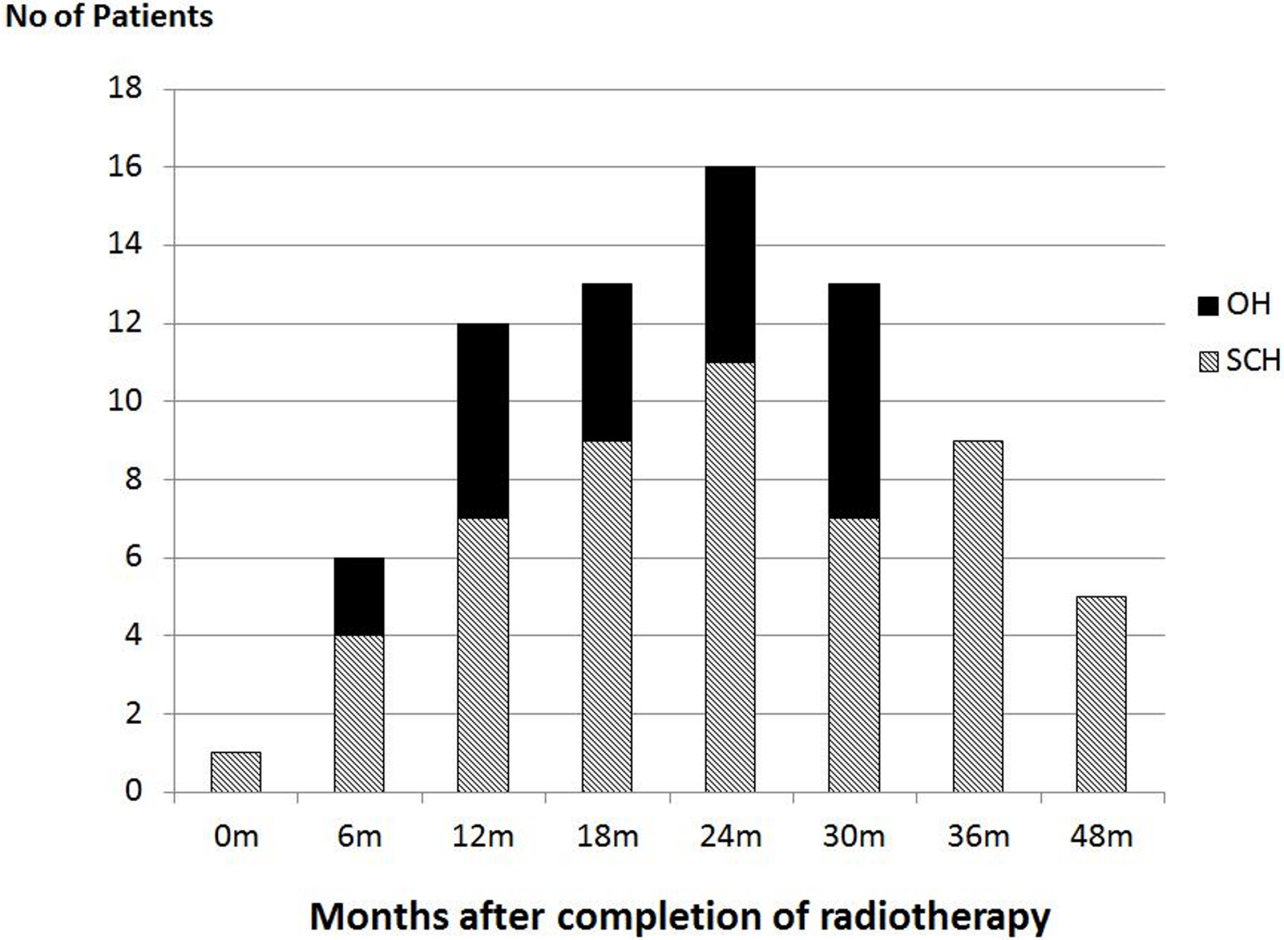
Hypothyroidism incidence from pre-radiotherapy to 48 months. 0m: Pre-radiotherapy, m = month, OH = overt hypothyroidism, SCH = subclinical hypothyroidism.

In the post-radiotherapy assessments, the incidence of overall hypothyroidism increased from 6 months (10.7%), then arrived at a peak at 24 months (28.6%) and dropped below 16.0% after 36 months (Fig 3). Majority of the hypothyroidism cases belonged to the subclinical type. The incidences of overt hypothyroidism were between 2.6% and 10.7% (black bars in Fig 3), which were found between 6 and 30 months intervals. No patient showed obvious clinical signs of hypothyroidism that required medical intervention.

In the correlation study, higher thyroid D_mean_ and D_50_ were significantly correlated with hypothyroidism incidence in 12 to 30 months (e.g. ρ > 0.40, p < 0.05 for D_mean_). When comparing between patients with hypothyroidism and no hypothyroidism between the 12 to 30 months post-radiotherapy intervals, the average thyroid D_mean_ and D_50_ were significantly higher in the patients with hypothyroidism (differences = 5.15 Gy and 6.65 Gy, p = 0.015 and 0.012 respectively) In addition, 81.8% of the hypothyroidism cases demonstrated thyroid D_mean_ of over 43 Gy.

## Discussion

This was first study that carried out close monitoring of the thyroid gland in 6-month interval for 48 months after radiotherapy. Our results showed that most of the thyroid gland changes occurred in the first 36 months after radiotherapy, and the thyroid volume and function reached a relatively steady state afterward.

With regard to the thyroid gland size, that there was dramatic loss of thyroid volume of nearly 30% in the first 12 months after radiotherapy in NPC patients. The trend went on with a slower rate for another 12 months before it became relatively steady. Furthermore, partial recovery in terms of gland volume was noted after the 30 month interval. The volume reduction of the thyroid was mainly due to the radiation induced damage of thyroid cells and blood vessels due to thyroiditis [3,15] which could be related with autoimmune reactions [16]. Besides, our study also demonstrated that the level of thyroxine (fT4) followed a similar trend as the thyroid volume change (Figs 1 and 2) implying that the production of the thyroxine was related to the shrinkage of the thyroid gland. This also implied that the thyroid volume size could be an indication of the thyroid function.

At the same time, the TSH level rose gradually after the completion of radiotherapy up to 24 months before taking a decreasing trend. This was because when the fT4 decreased in the body, based on the feedback mechanism, the pituitary gland secreted more TSH to stimulate the thyroid gland to produce more thyroxine. It was found that it had taken about 2 years to reach the balance before the TSH level started to drop and became steady. The reason of such relatively long recovery time was probably because the restoration of thyroxine required sufficient active thyroid cells. Therefore if the thyroid volume was still shrinking, even with the increased TSH, the thyroxine production rate could not be fully restored. In addition, our study also revealed that the thyroid gland function was partially recovered at around 30 months after radiotherapy in NPC patients when the thyroid gland started to increase in size. Up to now, except for a study on the treatment of Graves’ disease by iodine 131[17], there has been little report on the recovery of thyroid gland after radiotherapy and the mechanism involved is unknown. It is our plan to continue with longer follow up period for these patients to evaluate if the thyroid gland function can be completed recovered with time.

Due to the shrinkage of the thyroid gland and reduction of the thyroxine level after the completion of radiotherapy, it was expected that the cases of hypothyroidism were increased. The highest occurrence was around 24 months with an incidence rate of 28.6%. Such incidence was similar to some previous reports [18,19]. whereas a similar study by Fujiwara et al [20] on head and neck cancers reported a peak incidence of 36.4% at 24 months (median follow up period). In our study, most of the hypothyroidism cases were only detected by the blood test as they were either symptomless or presented with very mild symptom. Our results showed that higher mean thyroid dose was associated with increased incidence of hypothyroidism, and their correlations were significant in between 12 and 30 months post-radiotherapy period. This was reflected by the significantly higher mean thyroid D_mean_ and D_50_ of over 5 Gy in the hypothyroidism patients compared with the non-hypothyroidism group. Echoed with the comment made by Berges et al [19] which stated that the onset of hypothyroidism depends on the total dose delivered to the gland and the irradiated volume, keeping a reasonably low mean thyroid dose in radiotherapy planning could reduce the risk of thyroid complication. Since our study revealed that over 80% of the hypothyroidism patients received mean thyroid dose of over 43 Gy, this could serve as a reference for setting the tolerance dose of this organ in treatment planning. Our study echoed the results of the longitudinal studies conducted by Zhai et al [21] and Sommat et al [22] that they suggested thyroid mean dose constraint was approximately 45 Gy and V40 was a predictive parameter of primary hypothyroidism respectively, despite the study by Fujiwara et al [20] suggested a mean thyroid dose of 30 Gy as the threshold for predicting post-radiotherapy hypothyroidism. For this cohort of patients under current local practice, when no constraint was applied to the thyroid gland in radiotherapy planning, the mean thyroid D_mean_ was 43.3 Gy, which was marginally above this recommended tolerance. Therefore it was suggested the thyroid gland should be included as one of the organ at risks in the treatment planning of NPC so as to reduce the chance of post-radiotherapy hypothyroidism. Similar philosophy was supported by the report from Feen [23], who stated that the size of thyroid gland and radiation dose to the gland were key factors in the development of radiation induced hypothyroidism in head and neck cancer.

## Conclusions

Radiation induced hypothyroidism affected about 30% of the NPC patients after radiotherapy. The patterns of radiation induced reduction of thyroid volume and fT4 level were similar, in which both of them showed a decreasing trend from 6 to 30 months after completion of radiotherapy followed by partial recovery. The thyroid volume and function reached a relatively steady state after 36 months. The incidence of hypothyroidism also increased with time after radiotherapy up to 24 months post-radiotherapy and its frequency was associated with radiation dose delivered to the gland.
